# Progressive Pattern Interleaver with Multi-Carrier Modulation Schemes and Iterative Multi-User Detection in IoT 6G Environments with Multipath Channels

**DOI:** 10.3390/s24113648

**Published:** 2024-06-04

**Authors:** Shivani Dixit, Varun Shukla, Manoj Kumar Misra, Jose M. Jimenez, Jaime Lloret

**Affiliations:** 1 Department of Electronics & Communication, Pranveer Singh Institute of Technology, Kanpur 208001, India; shivani_badoni@rediffmail.com; 2 Department of Electronics & Communication, Allenhouse Institute of Technology, Kanpur 208001, India; varun.shuklaa@gmail.com; 3 Department of Computer Science, Allenhouse Institute of Technology, Kanpur 208001, India; manoj_misra12@rediffmail.com; 4 Instituto de Investigación para la Gestión Integrada de Zonas Costeras (IGIC), Universitat Politècnica de València, Grao de Gandia, 46730 Valencia, Spain; jojiher@dcom.upv.es

**Keywords:** 6G, IoT, NOMA, SC-FDMA, OFDM, IDMA, multiple access, ISI, multi-carrier modulation, multipath channel

## Abstract

Sixth-generation (6G) wireless networks demand a more efficient implementation of non-orthogonal multiple access (NOMA) schemes for severe multipath fading environments to serve multiple users. Using non-orthogonal multiple access (NOMA) schemes in IoT 6G networks is a promising solution to allow multiple users to share the same spectral and temporal resource, increasing spectral efficiency and improving the network’s capacity. In this work, we have evaluated the performance of a novel progressive pattern interleaver (PPI) employed to distinguish the users in interleaved division multiple access (IDMA) schemes, suggested by 3GPP guidelines as a NOMA scheme, with two multi-carrier modulation schemes known as single-carrier frequency-division multiple access (SC-FDMA) and orthogonal frequency-division multiplexing (OFDM), resulting in SC-FDMA-IDMA and OFDM-IDMA schemes. Both schemes are multi-carrier schemes with orthogonal sub-carriers to deal against inter-symbol interference (ISI) and orthogonal interleavers for the simultaneous access of multiple users. It has been suggested through simulation outcomes that PPI performance is adequate with SC-FDMA-IDMA and OFDM-IDMA schemes in terms of bit error rate (BER) under multipath channel conditions. Moreover, regarding bandwidth requirement and the implementation complexity of the transmitted interleaver structure, PPI is superior to the conventional random interleaver (RI).

## 1. Introduction

**Figure 1:**
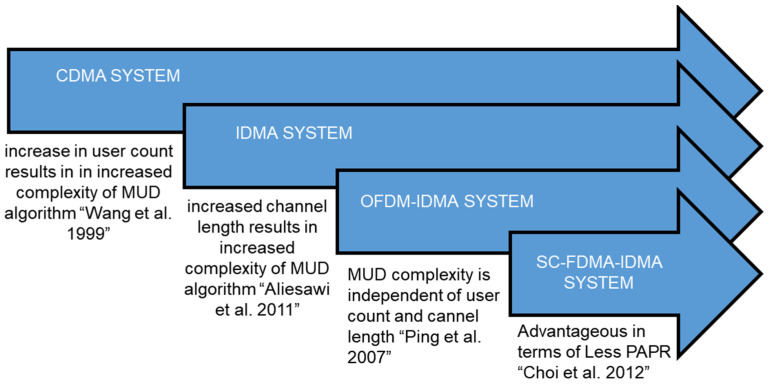
Advancement in multiple access techniques [8,12,18,19].

The exponential growth of IoT devices connected to a 6G network has led to an increased demand for non-orthogonal multiple access (NOMA) to efficiently share a limited spectrum, along with robust modulation schemes to cope with the multipath conditions of communication channels. The necessity to enhance performance through an innovative progressive pattern interleaver (PPI) utilized for user distinction in interleaved division multiple access (IDMA) schemes, recommended by the 3GPP guidelines as a NOMA scheme for 6G and IoT networks, arises from several pivotal factors in the development and evolution of wireless communication networks. These factors include the scarcity of spectral and energy resources, the exponential proliferation of IoT devices, and the surge in data traffic within 6G networks, where spectrum is a finite and valuable resource. It is imperative to enhance spectral efficiency to accommodate a larger number of IoT devices and deliver higher-quality services, given the growing demand for new applications and services requiring increased capacity. Consequently, the development of more efficient multiple access schemes capable of meeting these demands becomes essential. Signal fading resulting from multipath channels, coupled with the presence of numerous users in bandwidth-constrained networks, exacerbates the challenge of handling IoT scenarios [1,2]. NOMA emerges as a solution [3,4,5] to achieve spectral and power-efficient networks in multi-user environments. Figure 1 illustrates the evolution of various NOMA schemes along with their limitations. Spread-spectrum multiple access, in the form of code-division multiple access (CDMA) [6,7], was adeptly implemented in 3G networks to address fading channel communication scenarios. CDMA multi-user communication employs user-specific spreading codes to enable simultaneous user access. Multi-user detection (MUD) for CDMA signals is relatively straightforward to implement for a smaller number of users; however, as the user count increases in a network, the implementation complexity of CDMA-MUD also escalates exponentially [8]. Interleaved division multiple access (IDMA), proposed as a NOMA technique [1,4], can also serve as a viable option to facilitate multiple access in fading channel environments. In multiple access scenarios, the CDMA scheme assigns a unique spreading code to each user, whereas the IDMA scheme employs a unique interleaving pattern. Chip-level interleaving in the IDMA scheme results in less complex MUD [9,10,11] with faster convergence for the required bit error rate (BER) outcomes compared to CDMA. Consequently, IDMA emerges as a favorable choice for future generations of networks.

The IDMA scheme demonstrates superior and swifter convergence performance compared to the CDMA scheme; nonetheless, a severe multipath environment necessitates a more complex IDMA receiver with an increased number of paths [12]. Addressing this challenge, OFDM [13] has been integrated into 4G and 5G networks as a signal processing technique, offering significant resilience against multipath fading environments. Other authors such as those of [14] highlight the adaptability, performance evaluation and signal-processing capabilities of the OFDM system in DSP Platform for Various Purposes and Applications.

Single-carrier modulation systems are not particularly effective in multipath channel environments, as receiver complexity increases with the number of paths in the channel. In contrast, multi-carrier orthogonal frequency-division multiplexing (OFDM) divides the entire channel bandwidth into orthogonal multiple narrow sub-channels. These narrow orthogonal sub-channels encounter nearly flat fading, which can be addressed with relatively simple frequency domain equalization (FDE) using a single tap. The required frequency domain equalization operation in a multi-carrier OFDM system is facilitated by fast Fourier transform (FFT) and inverse fast Fourier transform (IFFT) operations.

Orthogonal frequency-division multiple access (OFDMA) [15] is employed in 5G networks to achieve the advantage of multi-carrier OFDM signals as multiple access technique; however, it also has limitations due to its high-peak power fluctuations. The SC-FDMA scheme [16] is another variant of the multi-carrier scheme to deal with multipath fading channels. Additional FFT operation in SC-FDMA spreads the single symbol over all the sub-carriers resulting in a low peak-to-average power ratio (PAPR) [17] structure. OFDMA and SC-FDMA are the multi-carrier orthogonal multiple access (OMA) methods suggested in 4G and 5G networks. For future generations of networks, these OMA schemes can be converted in to NOMA schemes with the integration of IDMA resulting in OFDM-IDMA [18] and SC-FDMA-IDMA [19,20,21,22,23,24] schemes. Distinguished orthogonal interleavers [25,26,27] in IDMA allow the overlapped sharing of bandwidth while keeping the multi-carrier nature of the signal to deal with multipath fading of the channel.

As stated, any IDMA-based multiple access system depends upon orthogonal interleavers to distinguish the simultaneous users. Various orthogonal interleavers suggested in the literature provide multiple access to the users in any IDMA-based NOMA system. This work analyzes the performance of progressive pattern interleaver with SC-FDMA-IDMA and OFDM-IDMA schemes. The performance of both multi-carrier NOMA schemes with PPI is analyzed and compared under multipath fading channel conditions to justify the possible implementation of the interleaver in next generation’s networks.

The rest of the paper is structured as follows. Section 2 presents some previous and related works. Section 3 offers an overall description of our proposed system model. Section 4 presents the simulation parameters and results. Section 5 explains the conclusion and future works.

## 2. Related Work

In this section, we summarize some previous works related to our proposal.

Some authors have studied and presented works on the application of NOMA technology to 6G networks. The authors of [28] explore the future vision and research opportunities for next-generation multiple access, focusing on the promising NOMA technology. According to these authors, NOMA’s main benefits to 6G networks are improved spectral efficiency, enhanced connectivity, low latency and reliability, flexibility in design, and improved user experience. Other authors, such as those of [29], explore the challenges faced by next-generation wireless networks and the development of advanced multiple access schemes to address them, focusing on the transition from NOMA to next-generation multiple access (NGMA). According to the work presented, NOMA’s ability to support non-orthogonal transmission, increase spectral efficiency, facilitate massive connectivity, ensure user fairness, and serve as a basis for further research makes it a key player in the evolution of NGMA for next-generation wireless networks. The authors of [30] explore how non-orthogonal multiple access (NOMA) and cognitive radio (CR) can improve spectrum-sharing performance in integrated satellite–terrestrial communication networks (ISTCNs), enabling high-speed and pervasive network access. In the work presented, they announce that the fusion of non-orthogonal multiple access (NOMA) and cognitive radio (CR) in CR-NOMA enhances the spectrum access of integrated satellite-terrestrial communication networks (ISTCNs) in the following ways: full spectrum access, interference suppression, efficient resource allocation, and improved decoding performance. In [22], the authors present an overview and outlook on how SCMA can enable massive connectivity in future 6G networks, paving the way for a ubiquitous intelligent mobile world. According to the work presented, in a massively distributed access system with NOMA, the key components are as follows: a multiple access scheme that allows multiple users to share the same time-frequency resource, enhances spectral efficiency and connectivity by serving multiple users simultaneously, and that can be integrated into the architecture of massively distributed access systems to support massive connectivity.

Other authors have studied the relationship of 6G and NOMA with energy consumption. Authors such as those of [31] propose the EPA scheme that optimizes power allocation, improves system capacity, enhances error performance, ensures fairness, and introduces a novel metric for evaluating energy efficiency in NOMA-based VLC systems, making it a promising approach for green IoT in 6G networks. Ishan et al. [32] presents a cutting-edge framework for optimizing energy efficiency in wireless networks using intelligent reflective surfaces and non-orthogonal multiple access beamforming. Their optimization process involves two stages: optimization of active beamforming and power allocation at the transmitter (BS), and optimization of passive beamforming at the intelligent reflective surface (IRS). Authors such as Jiang et al. [33] provide an overview of channel models’ importance in theoretical analysis, performance evaluation, and system deployment. It also discusses the advancements and challenges in modeling 6G wireless channels. They report that high-mobility and multiple mobilities in next-generation communication systems necessitate channel modeling that accounts for the non-stationary nature of channels in space, time, and frequency domains. Understanding these dynamic characteristics is crucial for designing accurate and efficient wireless communication systems for 6G networks.

In addition, some authors have studied and presented works related to progressive pattern interleavers. Authors such as those of [26] introduce an innovative method for generating an orthogonal interleaver set to support a large increase in user count while reducing implementation complexity and memory requirements. The proposed scheme enhances security and uniqueness for individual users in the system while maintaining optimal performance. Thet et al. [34] present a detailed investigation into how mutual coupling affects the achievable sum rate of NOMA systems in both uplink and downlink transmissions. The study focuses on a single-antenna-user NOMA system with directional beamforming in the millimeter wave channel. Other authors use progressive frequency-interleaved to improve the quality of the images. Wang et al. [35] present a detailed study on a novel progressive frequency-interleaved network (PFIN) developed for improving the quality of underwater images. The researchers have proposed a unique approach that combines the progressive frequency-domain module (PFDM) and convolution-guided module (CGM) to address the challenges of color distortion, low contrast, and blurred details commonly found in underwater images.

Some authors have presented surveys on grant-free non-orthogonal multiple access for IoT. Shahab et al. [36], in their survey, explore the challenges and solutions for enabling connectivity of a massive number of Internet of Things (IoT) devices in the fifth generation of wireless communications technologies. NOMA provides a promising solution for addressing the challenges of mMTC by enabling efficient resource utilization, grant-free access, improved spectral efficiency, and enhanced receiver design to support a massive number of IoT devices. Abbas [37] offers a thorough exploration of how visible light communication systems can leverage non-orthogonal multiple access techniques to improve spectral efficiency and capacity in wireless communication. He explores the client matching strategies, power allocation, and the applications of VLC-NOMA systems across different scenarios.

The results of our study significantly contribute to the development of more efficient NOMA schemes for future wireless networks in several key ways:

- Improved user identification: By utilizing techniques like progressive pattern interleaver (PPI), our study enhances the ability to identify users in congested network environments. This is crucial for ensuring efficient and reliable communication in future networks.
- Enhanced performance in fading channels: Our study demonstrates that NOMA schemes, when combined with PPI and multi-carrier modulation techniques like SC-FDMA-IDMA and OFDM-IDMA, can significantly improve performance in fading channels. This is essential for maintaining stable and high-quality communication in wireless environments.
- Optimized multi-carrier modulation: The optimization of multi-carrier modulation is key to maximizing spectral efficiency and network capacity. Our analysis of multi-carrier NOMA schemes with PPI provides insights into how these techniques can be optimized for future networks.
- Reduced implementation complexity: Simplifying the implementation of NOMA schemes, particularly with the use of techniques like PPI, can lead to reduced implementation complexity. This is important for ensuring the practicality and efficiency of wireless communication systems.

Our work builds upon previous studies by not only discussing the benefits of NOMA technology for next-generation networks but also providing concrete evidence of how implementing NOMA schemes with interleaving techniques can enhance network performance. By analyzing the performance of multi-carrier NOMA schemes with PPI, SC-FDMA-IDMA, and OFDM-IDMA, we offer justification for implementing interleaving in next-generation networks to improve overall performance and efficiency.

## 3. System Model

**Figure 2:**
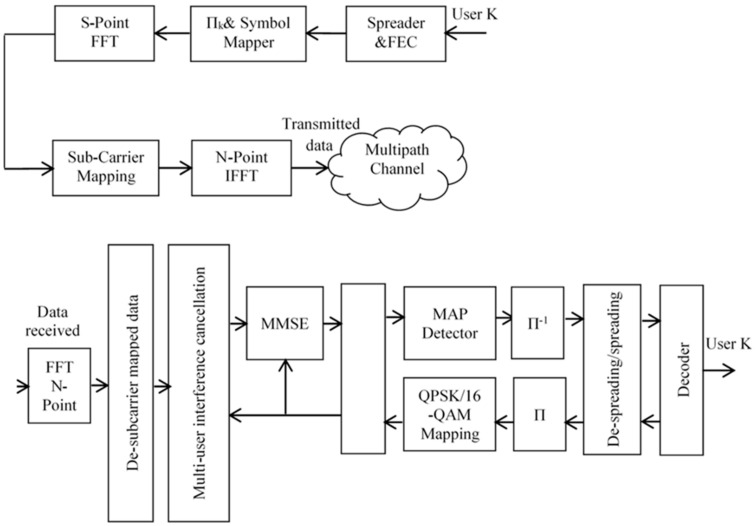
Transmitter and receiver structure of multi-carrier multiple access schemes.

This section elaborates on the system model. First, the structure of the transmitter is shown. Then, the algorithm of the PPI interleaver mechanism is described. Afterwards, the simulations on the channel considerations are shown, where delay profiles for multipath channel are presented. Finally, the messages of the structure depicted in Figure 2 are presented, and the communication process is discussed.

### 3.1. Transmitter Structure

The transmitter model for both multi-carrier schemes is depicted in Figure 2. The implementation of the multi-carrier modulation scheme is made feasible through FFT and IFFT operations. As per the transmitter structure illustrated, each user’s data undergo forward error correction (FEC), channel encoding, broadcasting, and user-specific interleaving to assign a unique identity to each user in a multiple access system. For coding, turbo codes or concatenated convolutional codes (CCC) are employed. According to the authors of [38], two primary types of coding exist in OFDM systems with turbo coding: turbo codes enhance system immunity to noise, while CCCs are utilized in turbo coding concatenation. After this FFT and IFFT operations are performed on a block size of S bits each. For OFDM-IDMA, FFT operation at the transmitter and corresponding IFFT at the receiver is not performed; the rest of the structure remains same as for SC-FDMA-IDMA [19,20].

For an individual user k, time-domain-modulated data symbols in a sequence of size S vector can be represented as follows:

(1) $$x_{k}={\left[x\left(0\right),\;x\left(1\right)\ldots x\left(S-1\right)\right]}^{T}$$

where (.)^T^ denotes transpose.

Time-domain-symbol sequence is frequency-spread using FFT of size S. FFT pre-coding is performed for SC-FDMA-IDMA signal only. This transformation results in a low PAPR of the SC-FDMA-IDMA system. Hence, each user of an SC-FDMA-IDMA or OFDM-IDMA system occupies S number of sub-carriers. Data symbols in the frequency domain can be represented as

(2) $$x_{k}={\left[X(0),X(1)\ldots X(S-1)\right]}^{T}$$

If N > S is the total number of orthogonal sub-carriers considered in a multi-carrier IDMA system design, then out of these N, S frequency domain data symbols are assigned to the S orthogonal sub-carriers for each user.

In these multi-carrier IDMA schemes, overlapped sub-carriers are allowed among users, since distinguished interleavers differentiate users in a multiple access system. Different orthogonal interleavers have been suggested in the literature to distinguish the users; here, we have implemented IDMA with the PPI interleaver suggested in [26]. Simple implementation complexity for the MUD and the least memory requirement is the reason for selecting the PPI interleaver to distinguish the users.

### 3.2. PPI Interleaver Mechanism

**Figure 3:**
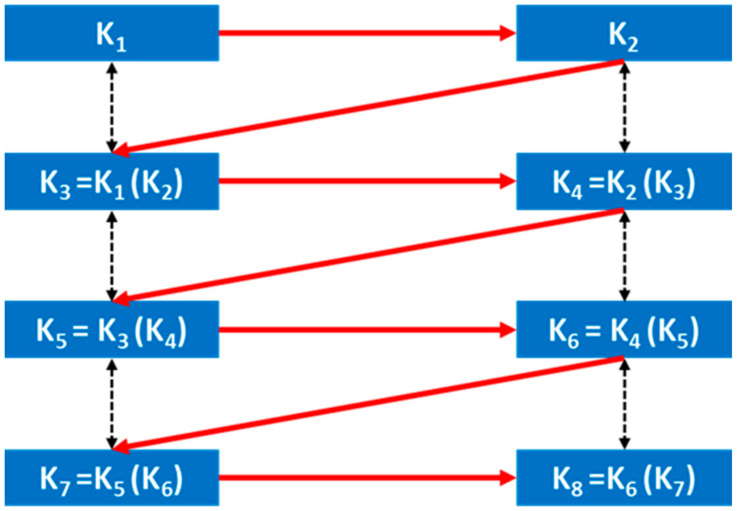
PPI interleaver generation.

As per the PPI method [26], shown in Figure 3, two mother interleavers K_1_ and K_2_ have been assigned to the first two users. The interleaving pattern for the third user is generated by interleaving the pattern of K_2_ with the pattern of K_1_, and the fourth user is assigned the pattern after interleaving the pattern of K_3_ with the pattern of K_2_. In sequence to this, to generate the patterns for the fifth and sixth users, patterns K_3_ and K_4_ work as mother interleavers. User K_5_ has been assigned the interleaving pattern after interleaving the pattern of K_4_ with the pattern of K_3_, and user K_6_ has been assigned the interleaving pattern after interleaving the pattern of K_5_ with the pattern of K_4_. Therefore, in this method, starting with two mother interleavers, successive interleaving patterns are generated, and the mother interleaving pattern changes continuously. This results in a relatively secure system in comparison to the power interleaver (PI) [39] suggested in the literature.

Sequence of PPI pattern generation at different stages in a communication system:

a. Sequence of base station interleaving pattern generation

First user is assigned with interleaving pattern K_1_ and second user is assigned with interleaving pattern K_2_. The third user is assigned with interleaving pattern K_3_ ≡ K_1_(K_2_), i.e., interleaving K_2_ pattern with the pattern of K_1_. The fourth user is given interleaving pattern K_4_ ≡ K_2_(K_3_), i.e., interleaving K_3_ pattern with the pattern of K_2_, and so on for the following users.

The first user is assigned interleaving pattern K_1_, and the second user is assigned interleaving pattern K_2_. The third user is allocated interleaving pattern K_3_, which is equivalent to interleaving pattern K_2_ with the pattern of K_1_. Similarly, the fourth user is assigned interleaving pattern K_4_, equivalent to interleaving pattern K_3_ with the pattern of K_2_, and so forth for subsequent users.

b. Sequence of mobile station interleaving pattern generation

Provided that for each value of n ≥ 1, interleaving patterns are transmitted for all 2n − 1 and 2n users, input the user number k and figure out ‘n’ such that 2n < k < 2n + 1. Following the above-mentioned algorithm at most 2n − 1 interleaving cycles are needed to generate interleaving pattern for the possible largest value of k.

At the mobile station, the interleaving pattern for each user is determined based on the user number assigned by the base station, minimizing the need for extensive information exchange. Additionally, a set of intermediate interleaving patterns is transmitted alongside primary interleavers to simplify computational tasks at the mobile station. This approach reduces the number of required interleaving cycles, especially when the base station broadcasts interleaving pattern generation methods for 2^n^ − 1 and 2^n^ users across all applicable values of n ≥ 1. For example, when considering a user k = 31, the calculation indicates that n equals 4, thus if interleaving patterns for (2^n^ − 1) and 2^n^ users (15 and 16) are available, a maximum of 2^n^ − 1 (15) additional cycles are required to compute the interleaving pattern for the 31st user.

c. Interleaving pattern generation during the implementation of MUD

The next user pattern generation depends upon the previous two user’s patterns and needs a single cycle only. For example, the interleaving pattern of K_3_ ≡ K_1_(K_2_) and interleaving pattern of K_4_ ≡ K_2_(K_3_).

The generation of the next user pattern depends on the patterns of the previous two users and requires only a single cycle. For instance, the interleaving pattern of K_3_ is derived as K_1_(K_2_), and the interleaving pattern of K_4_ is derived as K_2_ (K_3_).

Using the progressive pattern interleaving method reduces the memory requirements at each stage of the communication system compared to random interleaving (RI) and other interleavers discussed in the literature, with the added benefit of enhanced security. Overall, PPI surpasses other interleavers suggested in the literature in terms of bandwidth requirements and implementation complexity.

Following the interleaver assignment to each user, orthogonal sub-carrier mapping is implemented. Orthogonal sub-carriers can be mapped [40,41] in a localized manner or in a distributed manner. Distributed sub-carrier mapping distributes modulated symbols across the whole channel for a single user, whereas localized sub-carrier mapping assigns symbols in a contiguous manner for a single user. The corresponding multiple access schemes for SC-FDMA-IDMA system are termed as distributed frequency-division multiple access (DFDMA) and localized frequency-division multiple access (LFDMA). An appropriate sub-carrier mapping results in a frequency domain data vector as follows [19,20]:

(3) $$X_{k}={\left[X\left(0\right),\;X\left(1\right)\ldots X\left(N-1\right)\right]}^{T}$$

Here, for a particular user (N–S) orthogonal sub-carriers out of total N orthogonal sub-carriers have been assigned zero value, zero padding is applied.

Following this, frequency domain signal is transformed to time-domain signal through N point IFFT operation after implementing appropriate sub-carrier mapping. Further, the resulting time-domain signal is transmitted through a multipath channel.

### 3.3. Channel Considerations

In this work, the multipath channel has been simulated as the ITU Vehicular A [40,42] channel. Delay profiles for the multipath channel have been shown in Table 1.

### 3.4. Receiver Structure

The receiver system, as depicted in Figure 2, conducts iterative multi-user detection along with frequency domain equalization to detect the received signal [19,20]. The received signal is converted from the time to the frequency domain after undergoing an N-point FFT. De-subcarrier mapping is performed corresponding to the transmitter sub-carrier mapping. The received sequence in vectored form can be expressed as follows [19,20].

(4) $$Y=\sum_{\forall k}\sqrt{P_{k}}\wedge_{k}X_{k}+W$$

Here, P_k_ represents the assigned power to user k, ∧_k_ is the N × N matrix of channel frequency response for the kth user. Additionally, the de-subcarrier mapped data vector in the frequency domain is denoted by X_k_ as previously stated, along with *W* representing additive white gaussian noise (AWGN) with zero mean and variance σ_w_^2^.

Following this, MUD is performed iteratively with FDE to retrieve the individual signal of the users from the combined signal. Multi-user interference cancellation (MIC), minimum mean square error (MMSE) equalization, and decoding processes are performed successively for every cycle of iteration [19,20]. While detecting the signals for individual users, users’ signals are ordered according to their signal-to-noise ratio (SNR), with the detection of the highest SNR user first, followed by upcoming users’ detection as per their SNR value from high to low. Cancellation of the user signal with the highest SNR from the combined signal offers less multiple access interference (MAI) for the rest of the users. Therefore, the minimum MAI will be offered to the user having the lowest SNR, resulting in better overall performance.

Furthermore, minimum mean square error (MMSE) equalization is applied to all received symbols. To execute this, the mean $\bar{x_{k}}$ and variance v_k_ of the transmitted symbols for each user k can be expressed as [19,20].

(5) $$\bar{x_{k}}\left(n\right)≜E(\left(x_{k}\left(n\right)\right)=\sum_{r=0}^{2^{R}-1}\alpha_{r}.P(x_{k}\left(n\right)=\alpha_{r})$$

(6) $$v_{k}\left(n\right)≜Cov\left(x_{k}\left(n\right),x_{k}\left(n\right)\right)=\sum_{r=0}^{2^{R}-1}{\left|\alpha_{r}\right|}^{2}.P(x_{k}\left(n\right)=\alpha_{r})-{\left|\bar{x_{k}}\left(n\right)\right|}^{2}$$

Here, n represents all symbols ranging from 0 to S. Depending on the modulation order R, $\alpha_{r}$ denotes the corresponding modulation level, ranging from $\alpha_{0},\alpha_{1},\alpha_{2},\ldots \ldots ,\alpha_{2^{R}-1}$.

The probability P that a specific symbol has been assigned to a particular bit pattern is calculated as described in [19,20].

(7) $$P\left(x_{k}\left(n\right)=\alpha_{r}\right)=\prod_{l=0}^{R-1}\frac{1}{2}(1+{\overset{˘}{a}}_{r,l}.\tanh\left(L_{i}^{E}\left(b_{k}(Rn+l\right)\right)))$$

For each coded bit ${{\{b}_{k}(j)\}}_{j=0}^{RS-1}$, where j ranges from 0 to RS − 1, $a_{r,l}$ and ${\overset{˘}{a}}_{r,l}$ represent the inputs and outputs of the modulator, respectively, with the input to the modulator represented as 0 or 1 and the output from the modulator represented as 1 or −1.

For the outputs of the maximum a-posterior probability (MAP) decoder, the first iteration assumes $\bar{x_{k}}$ to be 0 and v_k_ to be 1. The signal $Z_{k}$ of a specific user for the mean vector $X_{k^{\prime }}$ can be obtained as follows [19,20]:

(8) $$Z_{k}=Y-\sum_{\begin{array}{c} k^{\prime }\neq \;k\\ k^{\prime }\in \;K \end{array}}\surd P_{k^{\prime }}\wedge_{k^{\prime }}\bar{X_{k^{\prime }}}$$

The result of MMSE equalization is the frequency domain estimate of equalized symbols as follows:

(9) $$\hat{X_{k}}=G_{k}^{H}Z_{k}-(G_{k}^{H}\wedge_{k}-\frac{1}{S}\sum_{s=0}^{S-1}(G_{k}^{H}\wedge_{k})\bar{{)X}_{k}}\sqrt{P_{k}}$$

where (.)^H^ denotes conjugate transpose. 

MMSE equalizer coefficients $G_{k}\left(s\right)$ at the sth sub-carrier are given by [19,20]

(10) $$G_{k}\left(s\right)=\frac{\sqrt{P_{k}}\wedge_{k}(s)}{\sum_{k^{\prime }=1}^{K}v_{k\prime }{\left|\wedge_{k^{\prime }}(s)\right|}^{2}+\sigma_{w}^{2}},\;\;\;\;fors=0,1,\ldots S-1$$

An IFFT operation is necessary to convert frequency domain symbols into sequential time-domain symbols before detecting the SC-FDMA-IDMA signal. The outputs of the IFFT, expressed as soft information log-likelihood ratios (LLRs) ${\left(L_{o}^{E}(b_{k}(j))\right)}_{j=0}^{2S-1}$, ranging from *j* = 0 to (2S − 1), are then passed to the de-interleaver and subsequently to the de-spreader. The soft outputs from the de-spreader, in terms of a priori LLRs ${\left(L_{i}^{D}(b_{k}(l))\right)}_{l=0}^{2S-1}$ from l = 0 to (2S − 1), are fed to the MAP decoder to compute the extrinsic information of the coded bits, as depicted in Figure 2. This output information is then looped back to the input of the MIC and MMSE equalizer as a priori information for the next iteration.

## 4. Simulation Parameters and Results

To evaluate the performance of progressive pattern interleaving (PPI) across both multi-carrier schemes, bit error rate (BER) performance has been simulated in both the Vehicular A channel environment, as discussed in the previous section, and the additive white gaussian noise (AWGN) channel environment. In these simulations, both localized frequency-division multiple access (LFDMA) and distributed frequency-division multiple access (DFDMA) sub-carrier mapping schemes have been employed for the SC-FDMA-IDMA scheme, while OFDM-IDMA simulations have utilized localized sub-carrier mapping. Additionally, all simulations have incorporated random interleaving (RI) for comparison with PPI across all multi-carrier multiple access scenarios. The MATLAB 9.4 (R2022b) [43] environment has been utilized for these simulations. In these simulations, BER performance has been plotted versus Eb/N0 (energy per bit to noise power spectral density ratio) in dB.

Simulation parameters have been set as follows unless stated specifically for individual instances. In NOMA-IDMA techniques, non-orthogonal multiple access has been provided through orthogonal PPI and RI interleavers in all these simulations. Orthogonal interleavers generated out of PPI interleaving [26] and RI interleaving have been assigned to different users to distinguish them from each other. Both the multi-carrier schemes have been implemented for N = 1024 number of sub-carrier system with convolutional coding of rate ½ on S = 128 number of symbols in sequence. Binary-phase-shift keying (BPSK)-modulated symbols of SC-FDMA-IDMA and OFDM-IDMA by spreading of length 8 have been simulated to plot BER performance of the schemes with PPI and RI interleaver. MUD has been implemented for 16 users and iteration count 5 has been considered for consolidated BER performance.

**Figure 4:**
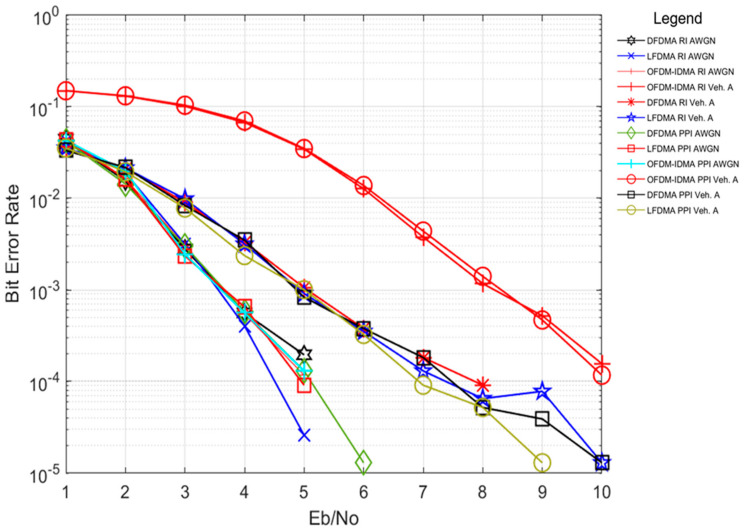
BER performance of PPI and RI with OFDM-IDMA, LFDMA, DFDMA in AWGN and Vehicular A (Veh. A) channels.

Simulation results shown in Figure 4 show the comparison of PPI and RI under AWGN and Vehicular A multipath channel environment with the mentioned multi-carrier multiple access schemes. The results reflect that the BER performance of PPI and RI for both multi-carrier schemes remains the same under AWGN channel condition. Addition of frequency diversity due to additional FFT operation in SC-FDMA-IDMA system results in better performance in comparison to OFDM-IDMA under Vehicular A multipath channel environment. Both sub-carrier mapping schemes including LFDMA and DFDMA result in the same BER performance for SC-FDMA-IDMA system with PPI and RI mechanism of interleaving. The above simulation results justify the appropriateness of PPI with suggested multi-carrier schemes under multipath channel conditions.

**Figure 5:**
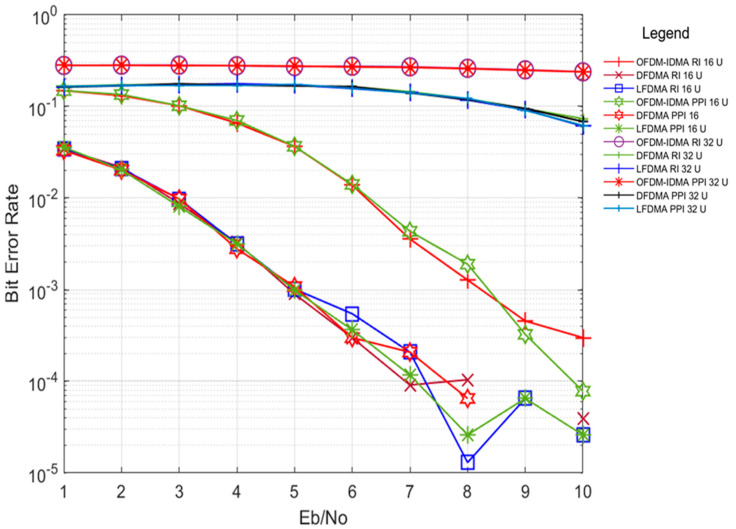
BER performance of PPI with OFDM-IDMA, LFDMA and DFDMA in Veh. A channel for user count (U) 16 and 32.

Figure 5 illustrates the performance comparison between progressive pattern interleaving (PPI) and random interleaving (RI) across both multi-carrier schemes in the Vehicular A channel environment, considering different numbers of users, namely 16 and 32. These simulations utilize PPI and RI interleaving techniques to differentiate between multiple users, while other parameters remain consistent with those previously specified. However, for a large number of users, both interleaving schemes struggle to maintain acceptable bit error rate (BER) performance in the multipath environment of the Vehicular A channel, attributed to the non-orthogonal multiple access (NOMA) access method employed in the system. To address this issue, channel scheduling algorithms [19] in conjunction with multi-carrier schemes yield improved performance. It can be concluded from results that PPI interleaver performs equally good as RI interleaver with large number of users also under multipath channel conditions.

**Figure 6:**
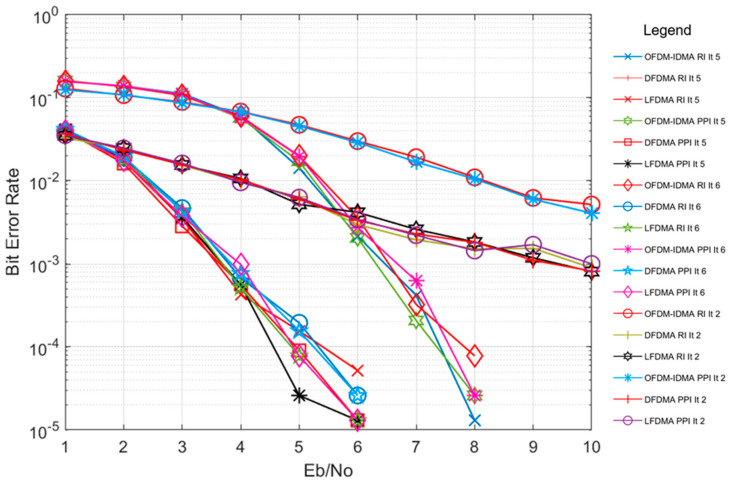
BER performance of PPI and RI for OFDM-IDMA, LFDMA and DFDMA in Veh. A channel for iteration count (It) 2, 5 and 6.

To gain further insight, simulations of PPI and RI have also been analyzed for multiple iteration counts with both multi-carrier schemes under a multipath scenario. Figure 6 shows the performance of both interleavers with the above-mentioned multiple access schemes for multiple iteration counts set at 2, 5, and 6 for MUD implementation at receiver. OFDM-IDMA performance is inferior to SC-FDMA-IDMA due to inherent frequency diversity in SC-FDMA-IDMA. Furthermore, it is observed that with iterative MUD implemented at the receiver of both schemes, an increase in iteration count improves the BER performance. This improvement saturates after a 5-iteration count, and further increase in iteration count simply increases the implementation complexity of the iterative MUD. Therefore, the optimum number of iteration counts has been considered as five in these simulations. Simulations conclude that PPI performs the same as RI under fewer iteration counts as well for the multipath channel environment.

**Figure 7:**
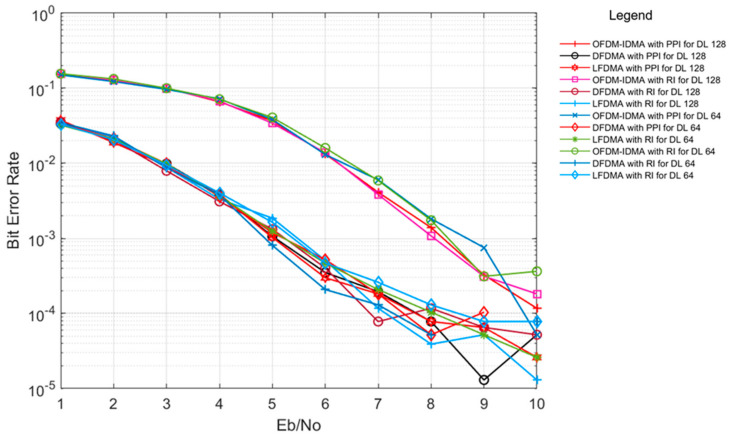
BER performance of PPI and RI for OFDM-IDMA, LFDMA and DFDMA in Veh. A channel for data length (DL) 128, 64.

Further, performance of PPI has been compared with RI under multipath channel conditions with above-mentioned multiple access schemes for variation in data length as shown in Figure 7. System with 16 users has been simulated for a 5-iteration count with the numbers of symbols considered as 128 and 64, respectively. Small data length shows more correlated interleaving patterns among users as compared to large data length. Therefore, results for small data length are inferior slightly as compared to large data length for both the interleaver types. It is evident from the results that PPI performs with the same efficiency as RI for smaller data lengths, even in a multipath channel environment.

In general, interleaving patterns for different users are less correlated if the data length is large; however, a small data length results in large correlation among patterns of different users resulting in inferior performance. Most of the orthogonal interleavers suggested in the literature [27] work well with large data lengths; however, due to their generation mechanism, if data lengths are small, the correlation among multiple users’ data increases, resulting in overall inferior performance of the NOMA receiver. PPI and RI work well with small data lengths. In comparison to RI, the generation of PPI is less complex while implementing MUD at the receiver. Therefore, PPI is a better choice to distinguish multiple users in NOMA scenario even with smaller data lengths.

## 5. Conclusions and Future Works

This work analyzes and compares the performance of progressive pattern interleaving (PPI) with random interleaving (RI) in conjunction with two multi-carrier schemes, namely SC-FDMA-IDMA and OFDM-IDMA, in both AWGN and multipath Vehicular A channel environments, aiming for potential non-orthogonal multiple access (NOMA) implementation in 5G/6G communication systems. The utilization of NOMA schemes within 6G and IoT networks is proposed as a promising solution to tackle various challenges, including spectral and energy resource scarcity, the exponential proliferation of devices and traffic, spectral efficiency enhancement, and the augmentation of network capacity to accommodate a large number of users and traffic simultaneously. In this context, NOMA has been integrated with PPI and RI for user discrimination. Upon evaluating the bit error rate (BER) performance of both interleaving techniques across multiple simulation scenarios, it is determined that PPI and RI exhibit comparable performance in AWGN environments across both multi-access schemes. However, in multipath Vehicular A channel environments, SC-FDMA-IDMA demonstrates superiority, while PPI demonstrates equivalent performance to RI with the considered multi-access schemes. The implementation of appropriate channel scheduling algorithms holds potential for enhancing system performance. Comparing the BER performance of PPI with RI under various scenarios leads to the conclusion that PPI performs favorably in terms of BER, with the additional advantage of lower implementation complexity compared to RI within multi-carrier schemes under multipath environments. Hence, it can be suggested that PPI is more appropriate choice for the implementation in a NOMA environment for a multipath channel scenario in future generation of networks. The rationale lies in the fact that, typically, PPI’s combination of comparable performance to RI in ideal conditions and effectiveness in challenging multipath environments, along with its lower implementation complexity, positions it as a more suitable option for NOMA implementation in future network generations, especially in scenarios involving multipath channels.

As a future work, we will use deep learning to enhance the error performance of uplink NOMA, to serve a great number of devices connected, in different environments. In addition, research and development will be further pursued in optimizing NOMA schemes for 6G and beyond, focusing on aspects such as energy efficiency, connectivity, latency and user fairness.

## Figures and Tables

**Table 1:** Delay profiles for multipath channel.

Channel Delay Profiles	Path-1 (Direct)	Path-2	Path-3	Path-4	Path-5	Path 6
Delay (in n-sec)	0	310	710	1090	1730	2510
Power (in dB)	0	−1.0	−9.0	−10.0	−15.0	−20.0

## Data Availability

Dataset available on request from the authors.
